# Evaluation of microbiological epidemiology and clinical characteristics of maternal bloodstream infection: a 10 years retrospective study

**DOI:** 10.3389/fmicb.2023.1332611

**Published:** 2024-01-08

**Authors:** Junfei Guo, Yongbing Wu, Huan Li, Wenyu Deng, Weiming Lai, Chunming Gu, Mingyong Luo

**Affiliations:** ^1^Clinical Laboratory Department, Guangdong Women and Children Hospital, Guangzhou, China; ^2^Information Department, Information Department, Guangdong Women and Children Hospital, Guangzhou, China

**Keywords:** maternal bacteremia, bloodstream infection, sepsis, infection, pregnancy

## Abstract

**Objective:**

Although the incidence of bloodstream infection (BSI) during pregnancy is relatively low, it can lead to unfavorable outcomes. The aim of our study was to analyze the clinical and microbiological characteristics of maternal bacteremia and to assess maternal and fetal outcomes.

**Methods:**

Our study was a retrospective study conducted in a tertiary women and children’s hospital in Guangzhou, China, from 2013 to 2022. Data were extracted from medical records and the laboratory information system. The participants were divided into groups, and the difference between the groups was analyzed.

**Results:**

The incidence of maternal BSI during the 10 years study period was 10.2 cases/10,000 maternities, with a peak found from 2014 to 2016. *Escherichia coli* (48%) was the predominant causative pathogen, followed by *Streptococcus agalactiae* (13%). Gestational diabetes mellitus (GDM) (15%) was the most common underlying condition among maternal BSI episodes. Urinary tract (13%) and genital tract (28%) were the predominant source of BSI. About 14% of neonates were infected, and BSI was the most common type of infection. *E. coli* was the predominant pathogen in mother-neonate pairs with concurrent BSI. Premature rupture of membranes (PROM, OR:4.68) and preterm birth (OR:3.98) were the risk factors predicting neonatal infection. More than 85% of the *E. coli* were resistant to ampicillin (AMP) and 50% of the *E. coli* were extended-spectrum β-lactamase (ESBL)-producing bacteria.

**Conclusion:**

Maternal BSI is a rare event, but continuous monitoring on the aspects of pathogen composition, antimicrobial resistance characteristics, and risk factors for adverse outcomes remains necessary to further reduce poor outcomes and mitigate bacterial resistance.

## Introduction

Bloodstream infection in pregnancy or during the perinatal period is still a significant clinical problem with high morbidity and mortality, even in developed countries (Waterstone et al., 2001; Surgers et al., 2013; Zou et al., 2021). There are some studies focusing on maternal infectious diseases in mainland China (Lin et al., 2021; Zou et al., 2021; Zhong et al., 2022) but only one published study focused on perinatal BSI could be found (Zou et al., 2021). Very little is known about various aspects of maternal bacteremia in relation to changes of microbiological epidemiology and obstetric outcomes. There are also few data available on the consequences of maternal bacteremia on the fetus. The pathogen composition and the antimicrobial resistance pattern of BSI-associated pathogens varies over time, so it is important to understand the prevalence of different types of microorganisms and their antimicrobial resistance characteristics.

To improve understanding of BSI during pregnancy or during the perinatal period, we conducted a retrospective study over a 10 years period in a tertiary women and children’s hospital in Guangzhou, China. We aimed to study. The current incidence and characteristics of BSI during pregnancy and the perinatal period, knowledge of which may improve the management of maternal BSI treatment and reduce adverse outcomes.

## Materials and methods

### Study population and setting

This was a retrospective study conducted in a tertiary women’s and children’s hospital in southern China. The Ethical Review Board of our hospital approved the study. All our research was conducted according to the relevant guidelines and regulations. Informed consent was obtained, and patients were given the opportunity to refuse to have their clinical records to be used for research. Patient privacy and confidentiality of data confidentiality were maintained in accordance with the Declaration of Helsinki.

### Definitions

The definition and source of bloodstream infections were based on the European Centre for Disease Prevention and Control case definitions. Antenatal maternal BSI was defined as BSI occurring during conception and onset of labor. Intrapartum BSI refers to BSI that occurred between the onset of labor and delivery of the placenta (48 h after delivery in most of our cases). Postpartum maternal BSI is defined as BSI occurring 42 days after delivery of the placenta. Co-infection was defined as a mother-neonate pair simultaneously infected with a bacterial pathogen (same or different) in our study.

### Data collection

Records of underlying conditions and outcomes, as well as demographic, clinical, and microbiological information, were extracted from the patient’s medical record and the laboratory information system. The following obstetric information was collected: mode of delivery, gestation, the situation of PROM, and intrauterine fetal distress (FIUD) status. The following neonatal information was collected: infection situation, ICU admission, and outcome. Evidence of fetal infection in the neonates was determined on the basis of clinical, microbiological, and histological findings. A positive blood culture report included organism identification and antibiotic susceptibility results. Organism identification was performed using MALDI-TOF and the antibiotic susceptibility using the Vitek2 compact system.

### Statistical analyses

Data were analyzed using SPSS 24.0 version. Frequencies and proportions were used to describe the data. Binary logistic regression analysis was used to analyze risk factors. Univariate logistic regression analysis was performed first; variables that showed a significant association in univariate logistic regression analysis were included in the multivariate logistic regression model. *p*-values <0.05 were considered to be statistically significant.

## Results

**Figure 1:**
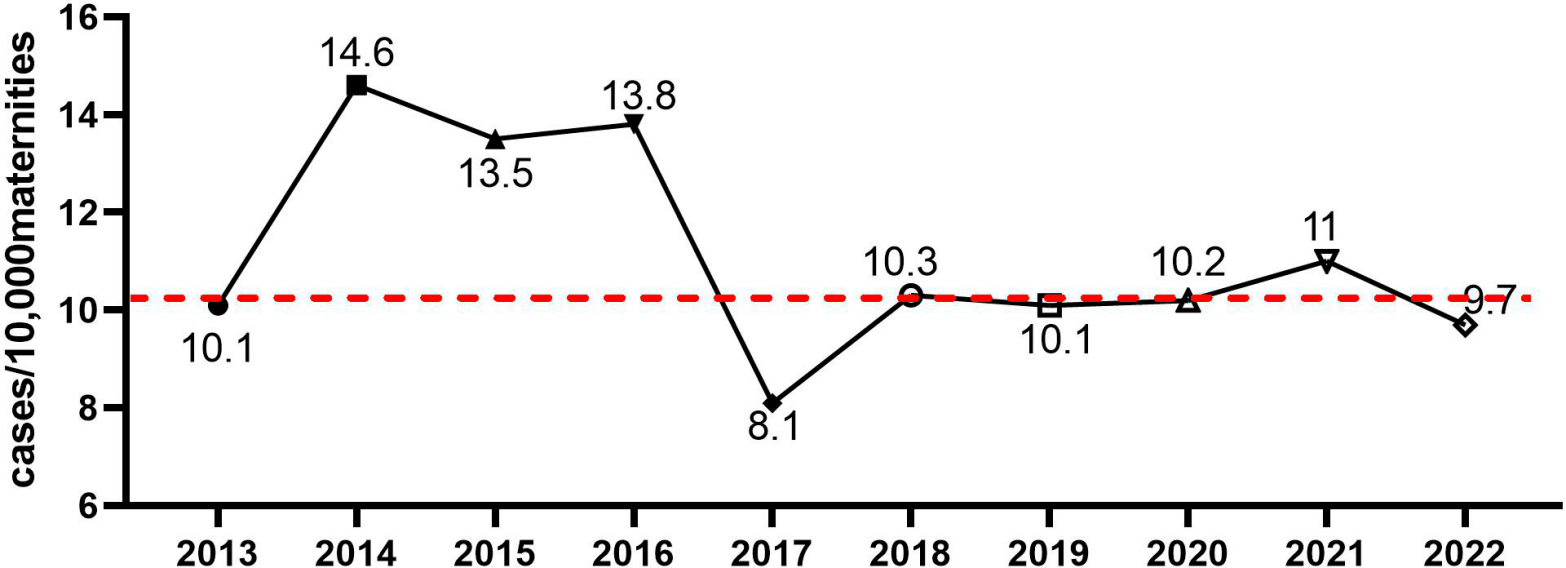
The incidence of maternal bacteremia from 2013 to 2022 in our institute. The incidence of maternal bacteremia was calculated, and the dashed line represented mean incidence across the 10 years period.

During the 10 years study period, a total 141,995 of pregnant women who delivered at our institute were included in the analysis. Laboratory-confirmed BSI was identified in 146 obstetric patients, resulting in a maternal BSI incidence of 10.28 cases/10,000 deliveries. As shown in Figure 1, a peak in maternal BSI incidence was found in 2014 and 2016, and the lowest incidence was found in 2017. Seventeen (12%) cases of maternal BSI occurred during the antenatal period; 77 (53%) cases of maternal BSI occurred during the intrapartum period; 52 (36%) maternal BSI cases occurred during the postpartum period. Two cases (1.4%) of maternal BSI were fatal, and 53 (36.3%) cases required admission to intensive care.

As shown in Table 1, 86 (58.9%) maternal BSI cases occurred in women with term delivery, and the distribution of gestational age was similar among maternal BSI cases occurring at different stages (antepartum, intrapartum, or postpartum period). The most common underlying condition seen was GDM (15%), while chronic hypertension, chronic kidney disease, and anemia were also reported as underlying conditions. Ninety-six (65.8%) cases had a caesarean delivery, 33 cases (22.6%) had a spontaneous vaginal delivery, and 10 cases (6.8%) had an operative vaginal delivery; the mode of delivery of the remaining seven episodes (4.8%) was not recorded. The percentage of caesarean deliveries was significantly higher in antepartum maternal BSIs compared with intrapartum or postpartum maternal BSIs.

**Table 1:** The clinical characteristics of maternal bacteremia occurring at different time points during the labor process.

Characteristic	Antepartum *n* (percent)	Intrapartum *n* (percent)	Postpartum *n* (percent)	Total *n* (percent)
**Gestational age, No.**				
≤28	5 (29.4%)	9 (11.7%)	9 (17.3%)	23 (15.8%)
28–35^+6^	6 (35.3%)	23 (29.4%)	8 (15.4%)	37 (25.3%)
≥36	6 (35.3%)	45 (58.4%)	35 (67.3%)	86 (58.9%)
**Underling conditions, No.**				
Diabetes	2 (11.8%)	10 (13%)	10 (19.2%)	22 (15.1%)
Chronic kidney disease	1 (5.9%)	0	0	1 (0.7%)
Chronic hypertension	1 (5.9%)	2 (2.6%)	3 (5.8%)	6 (4.1%)
Obesity	2 (11.8%)	0	0	2 (1.4%)
Anemia	0	2 (2.6%)	1 (1.9%)	3 (2.1%)
Other	3 (17.6%)	13 (16.9%)	9 (17.3%)	25 (17.1%)
**Site of infection, No.**				
Genital tract	3 (17.6%)	22 (28.6%)	17 (32.7%)	42 (28.8%)
Urinary tract	4 (23.5%)	7 (9.1%)	8 (15.4%)	19 (13%)
Wound	0	1 (1.3%)	0	1 (0.7%)
Respiratory	0	1 (1.3%)	0	1 (0.7%)
Other	0	3 (3.9%)	3 (5.8%)	6 (4.1%)
**Maternal outcome, No.**				
Survival	17 (100%)	75 (97.4%)	52 (100%)	144 (98.7%)
ICU admission	5 (29.4%)	31 (40.3%)	17 (32.7%)	53 (36.3%)
Death	0	2 (2.6%)	0	2 (1.4%)
**Fetal outcome, No.**				
Survival	13 (76.5%)	72 (93.5%)	49 (94.2%)	134 (91.7%)
Stillbirth	2 (11.8%)	2 (2.6%)	1 (1.9%)	5 (3.2%)
Preterm birth	11 (64.7%)	32 (41.6%)	12 (23.1%)	55 (37.7%)
Neonatal ICU admission	13 (76.5%)	71 (92.2%)	33 (63.5%)	117 (80.1%)
Bacteremia	2 (11.8%)	7 (9.1%)	2 (3.8%)	11 (7.5%)
Death	2 (11.8%)	3 (3.9%)	2 (3.8%)	7 (4.8%)
**Organism**				
*E. coli*	7 (41.2%)	31 (40.3%)	33 (63.5%)	71 (48.6%)
*K. pneumoniae*	1 (5.9%)	5 (6.5%)	5 (9.6%)	11 (7.5%)
Other G^−^ bacteria	3 (17.7%)	2 (2.6%)	3 (5.8%)	8 (5.5%)
*S. agalactiae*	2 (11.8%)	16 (20.8%)	1 (1.9%)	19 (13%)
*E. faecalis*	1 (5.9%)	2 (2.6%)	6 (11.5%)	9 (6.2%)
Other G^+^ bacteria	3 (17.7%)	16 (20.8%)	9 (11.7%)	28 (19.2%)
**Method of delivery, No.**				
Cesarean	13 (76.5%)	48 (62.3%)	35 (67.3%)	96 (65.8%)
Spontaneous vaginal	2 (11.7%)	19 (24.7%)	12 (23.1%)	33 (22.6%)
Operative vaginal	1 (5.9%)	9 (11.7%)	0	10 (6.8%)
Uncertain	1 (5.9%)	1 (1.3%)	5 (9.6%)	7 (4.8%)

Sixty-five (45%) maternal BSI episodes had definite sources of infection: 29 cases (19%) had genital tract infection, 18 cases (12%) had urinary tract infection, three cases had urinary tract and genital tract co-infection, and 11 cases (8%) had placental and amniotic fluid infection. The urinary tract was the cause of BSIs in 23%, 9%, and 15% of antepartum, intrapartum, and postpartum maternal BSIs, respectively. The genital tract was the source of BSIs of 17%, 28%, and 32% of antepartum, intrapartum, and postpartum maternal BSIs, respectively.

**Figure 2:**
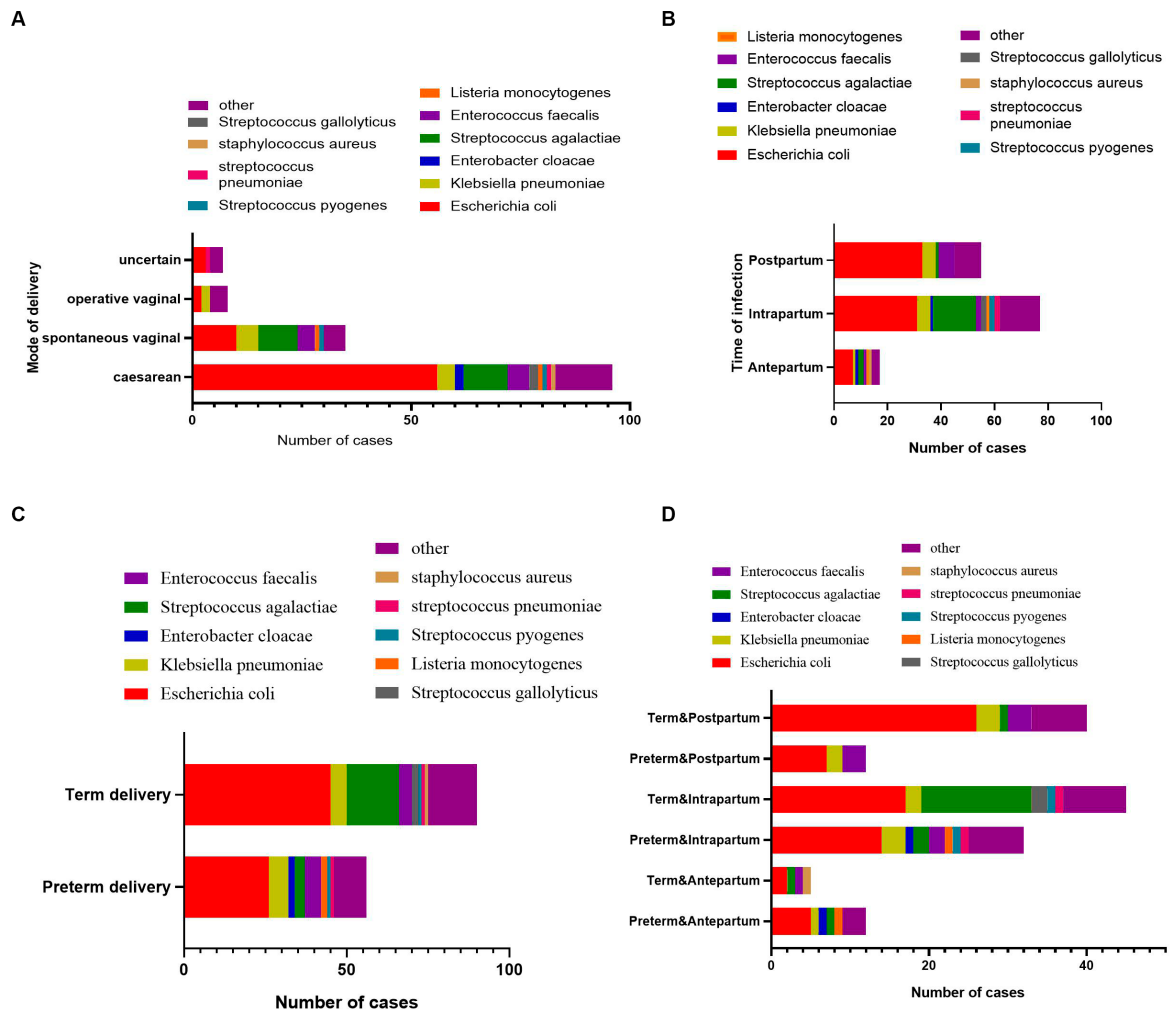
The pathogen composition of maternal bacteremia with different clinical characteristics. **(A)** The pathogen composition of maternal bacteremia with different delivery modes. **(B)** The pathogen composition of maternal bacteremia occurring at different time points during the labor process. **(C)** The pathogen composition of maternal bacteremia with term delivery or preterm delivery. **(D)** The pathogen composition of maternal bacteremia with term delivery or preterm delivery at different time points during the labor process.

As shown in Table 1, *Escherichia coli* (71 isolates, 48.6%) was the predominant causative pathogen in maternal BSI cases: 41% of antenatal, 40% of intrapartum, and 63% of postpartum maternal BSI episodes were caused by *E. coli*. *Streptococcus agalactiae* (19 isolates, 13%) was the second most common pathogen, accounting for 11.8%, 20.8%, and 1.9% of antenatal, intrapartum, and postpartum cases, respectively. *Klebsiella pneumoniae* and *Enterococcus faecalis* ranked the third and the fourth most common causative pathogen, respectively. The pathogen composition of maternal BSIs was similar in cases with different modes of delivery (Figure 2A), but the pathogen composition was different in terms of time point of BSI (antepartum, intrapartum, or postpartum period). Almost all the *S. agalactiae* strains were separated from mothers with BSI in the intrapartum period (Figure 2B). As shown in Figures 2C,D *S. agalactiae* strains were mainly isolated from mothers with term delivery and intrapartum BSI; *E. coli* was the main causative pathogen of BSI cases with term delivery and postpartum BSI or those with preterm delivery and intrapartum BSI.

In our study population, two women died from BSI caused by *Streptococcus pyogenes* and *E. coli*, respectively. Fifty-three (36%) women required admission to the intensive care unit. All women involved were singletons, and the overall neonatal in-hospital survival rate of the newborns was 91.7%. The overall stillbirth rate was 3.2%, and 55 newborns (37.7%) were preterm. Eighty percent of the neonates required admission to neonatal ICU. Eleven (7.5%) neonates had BSI and seven neonates died before discharge.

Twenty-one (14.3%) neonates were infected, and the predominant site of infection was the blood stream. *E. coli* was the predominant pathogen in neonates. Among 21 mother-neonate pairs with co-infection, 15 pairs were infected by the same pathogens: nine mother-neonate pairs had BSI caused by *E. coli*, two mother-neonate pairs had BSI caused by *Streptococcus agalactiae*, two mother-neonate pairs had BSI caused by *K. pneumoniae*, and one mother-neonate pair had BSI caused by *Listeria monocytogenes* and *Streptococcus mitis*, respectively. As shown in Table 2, preterm birth and PROM were the risk factors predicting whether the newborns of women with maternal BSI had BSI. Maternal age, pyrexia, cesarean section, and FIUD were not risk factors for predicting neonatal BSI.

**Table 2:** The logistic analysis of risk factors to predicted neonatal infection.

Items	*β*	SE	*χ*2	*p*	OR	95% CI
Age	0.077	0.055	1.97	0.16	1.08	0.97–1.203
PROM	1.543	0.533	8.374	0.004	4.68	1.645–13.312
FIUD	0.51	0.565	0.814	0.367	1.665	0.55–5.038
Premature delivery	1.381	0.529	6.813	0.009	3.98	1.411–11.229
Mother with pyrexia	−0.011	0.627	0	0.986	0.989	0.29–3.379
Cesarean	−0.1	0.567	0.031	0.86	0.905	0.298–2.752
Dirty amniotic fluid	0.654	0.648	1.018	0.313	1.923	0.54–6.849

**Figure 3:**
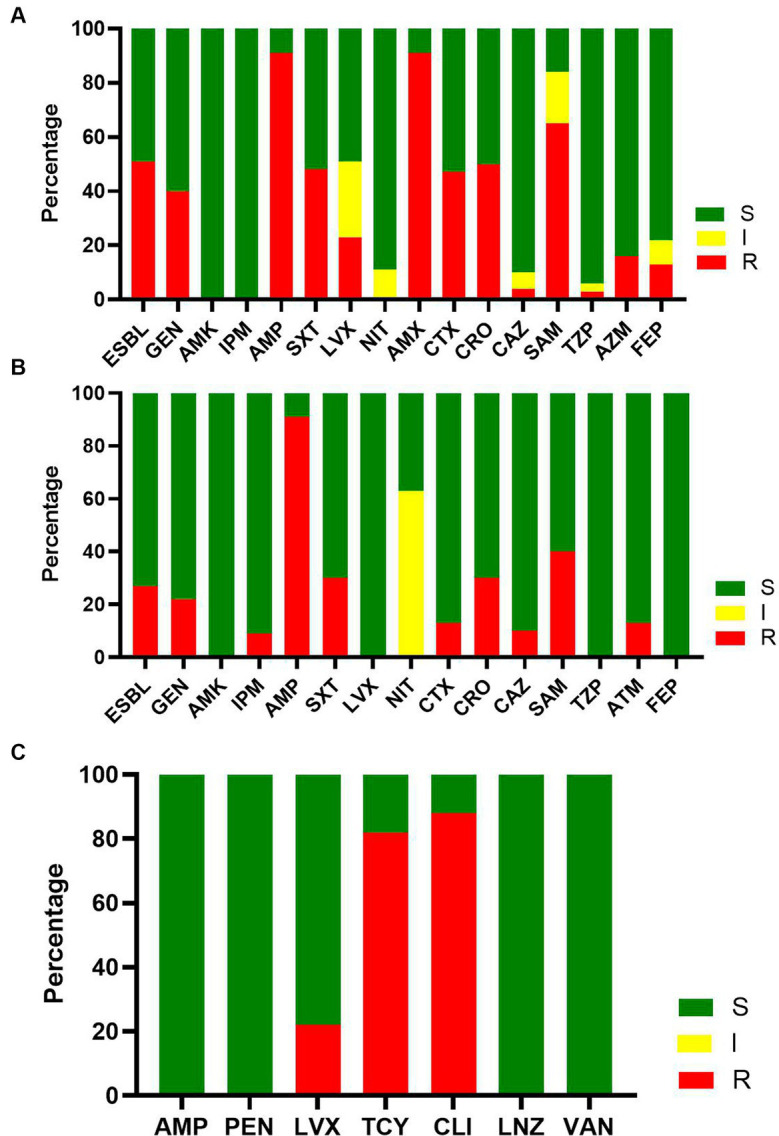
The susceptibility result of major pathogens of maternal bacteremia. **(A)** The susceptibility result of *E. coli* to commonly used antimicrobials. **(B)** The susceptibility result of K. pneumonia to commonly used antimicrobials. **(C)** The susceptibility result of *S. agalactiae* to commonly used antimicrobials.

Forty-eight and six tenths percent of the maternal BSI was caused by *E. coli*, and 50% of the *E. coli* isolates were ESBL-producing strains (Figure 3A). Only one carbapenem-resistant *E. coli* strain was isolated. More than 80% of the *E. coli* isolates were resistant to AMP and amoxicillin and clavulanate (AMX); 65% of the *E. coli* isolates were resistant to ampicillin and sulbactam (SAM). All the *E. coli* isolates were susceptible to amikacin (AMK) and imipenem (IPM). More than 95% of the *E. coli* isolates were susceptible to ceftazidime (CAZ) and piperacillin tazobactam (TZP).

Seven and five tenths percent of maternal BSIs were caused by *K. pneumoniae*, and 25% of *K. pneumoniae* isolates were ESBL-producing *K. pneumoniae* (Figure 3B). Only one carbapenem-resistant *K. pneumoniae* was isolated. More than 80% of the isolated *K. pneumoniae* were susceptible to AMP. All the isolated *K. pneumoniae* were sensitive to AMK, levofloxacin (LVX), TZP, and cefepime (FEP).

*S. agalactiae* was the dominant Gram-positive pathogen isolated (13%). More than 80% of *S. agalactiae* isolates were resistant to tetracycline (TCY) and clindamycin (CLI), and all the *S. agalactiae* isolates were susceptible to AMP, penicillin (PEN), linezolid (LNZ), and vancomycin (VAN) (Figure 3C).

## Discussion

The incidence of maternal BSI was approximately 10 cases/10,000 maternities, meaning it remains a rare occurrence in pregnancy and is relatively lower than previously reported levels (Waterstone et al., 2001; Surgers et al., 2013; Acosta et al., 2014; Hensley et al., 2019; Chen et al., 2021). A study focusing on maternal sepsis in Guangzhou city reported an incidence of 79.35/10,000 (Lin et al., 2021), significantly higher than our study. Sepsis is a life-threatening organ dysfunction caused by dysregulated host response to infection (Singer et al., 2016), and BSI represents only one type of sepsis-related infection. This may explain the above-described difference between the study by Lin et al. (2021) and our own, although both were conducted in the same city. Improved hygiene conditions and widespread screening for *S. agalactiae* carriage and application of per-partum prophylaxis (Koenig and Keenan, 2009) may explain the decline in BSI incidence. However, *S. agalactiae* accounted for 13% of our BSI episodes, similarly to previously reported data (Deutscher et al., 2011; Surgers et al., 2013). Boyer and Gotoff (1986) and Deutscher et al. (2011) found that per-partum prophylaxis in pregnancies with a positive *S. agalactiae* screen could significantly reduce early-onset neonatal *S. agalactiae* infection but had little effect on reducing maternal infection. This may explain the persistence of *S. agalactiae*-associated maternal infection even with a large initiation of screening for *S. agalactiae*.

Consistent with previous studies (Surgers et al., 2013; Zou et al., 2021; Ornaghi et al., 2022), *E. coli* was the predominant pathogen of maternal BSI. However, in studies conducted in African countries, *Salmonella typhi* and *Streptococcus pneumoniae* were the dominant pathogens in Malawi (Musicha et al., 2017), and *Staphylococcus aureus* and *K. pneumoniae* were the major pathogens in Rwanda (Habyarimana et al., 2021). We found that maternal BSIs occurring at different time points had different pathogen compositions; *S. agalactiae* was mainly isolated from intrapartum maternal BSI episodes, which is consistent with what was reported from a study in Ireland (Knowles et al., 2015). The proportions of *K. pneumoniae* and *E. faecalis* were higher than in other studies (Surgers et al., 2013; Knowles et al., 2015). This further supports the idea that the pathogen spectrum and pattern of antimicrobial resistance associated with BSIs often differ between affected regions, due to differences in epidemiological and geographical characteristics and the clinical use of antimicrobial drugs over time. We should take these into consideration when dealing with pregnancy with suspected BSI.

In our study, there were only six cases of maternal bacteremia caused by anaerobic bacteria, five of which were identified as *Bacteroides fragilis* and the remaining one as Clostridium species. The proportion of anaerobic bacteria was lower than that reported in other studies (Blanco et al., 1981; Surgers et al., 2013; Zou et al., 2021). The detailed reason for the lower rate of anaerobic bacteria is unclear, although different patient composition and hygiene levels and different antibacterial drug exposure levels may be part of the reason. Moreover, underlying conditions like obesity may play a role in different pathogens’ acquisition, as already described (Kankuri et al., 2003; Surgers et al., 2013). Although polymicrobial bacteremia was found in previous studies (Monif and Baer, 1976; Newton, 1993; Surgers et al., 2013), we did not observe any polymicrobial bacteremia in our study. All this indicates a change in the pathogenic epidemiology associated with maternal BSI and justifies the need to re-evaluate empirical antimicrobial regimens in pregnancy with suspected BSI.

The sources of infection in our study were mainly the urinary and genital tract; 39% of our cases had concomitant urinary tract or genital tract infection caused by the same pathogen as BSI. Chorioamnionitis has been reported as a source of bacteremia in several studies (Newton, 1993), but in our study we did not systematically perform a pathological examination of the placenta, and therefore we cannot assess the rates of chorioamnionitis.

Maternal BSI can lead to adverse fetal outcomes. There were five stillbirths and seven neonates who died before discharge, representing 8% of all the study patients, similar to the 10% mortality rate in Surgers’s study (Surgers et al., 2013). Thirty-seven percent of the neonates were born preterm and 80% of neonates born to mothers with BSI required admission to the ICU. Besides the tremendous social and family concerns following a newborn death, the high economic burden of medical care in this case further highlights the significance of infection control policies (IPCs) in order to eliminate maternal and neonatal BSIs. Fourteen percent of the neonates were infected and 7.5% neonates developed bacteremia in our study, which was significantly higher than that reported by Surgers et al. (2013). The higher preterm delivery rate in our study may be part of the reason for the higher neonatal infection; the preterm delivery rate in Surgers’s study was 29% (Surgers et al., 2013). In our study, PROM and preterm birth were risk factors for neonatal bacteraemia.

The high rates of antimicrobial resistance in our study accentuate the need for a drastic reduction of BSIs due to the lack of effective antibiotics that can be administered to pregnant women. In general, aminopenicillins have been considered as the first-line antimicrobial for empiric treatment of suspected BSI in pregnancy (Harris, 2023). However, in our study, more than 85% of *E. coli* and *K. pneumoniae* isolates were resistant to AMP and amoxicillin-clavulanate, and about 50% of *E. coli* isolates were ESBL-producing bacteria. As *E. coli* and *K. pneumoniae* account for more than half of maternal BSI episodes, it is important to reconsider the use of aminopenicillins as empirical antimicrobial therapy regimens in pregnant women. We believe that a third-generation cephalosporin may be a more appropriate first-line empiric agent in pregnant women with suspected BSI originating from urinary and genital tract infections.

Our study has some limitations. It described a single-center experience, so our results cannot be extrapolated at a regional or country level. Moreover, we did not include a control group of non-pregnant women. Molecular identification of resistant pathogens and investigation of a possible clonal spread was not performed. However, our study remains one of the biggest in the field with a thorough description of the incidence of maternal BSI, a rare but potentially lethal condition with dramatic consequences at personal, social, and healthcare levels. Therefore, further multicenter well-designed prospective studies could investigate epidemiological, clinical, economic, and laboratory aspects (including molecular resistance mechanisms) and serve as an important tool for the application of effective infection control policies.

## Data availability statement

The raw data supporting the conclusions of this article will be made available by the authors, without undue reservation.

## Ethics statement

The studies involving humans were approved by Ethical Review Board of Guangdong Women and Children’s Hospital approved the study. The studies were conducted in accordance with the local legislation and institutional requirements. Written informed consent for participation was not required from the participants or the participants’ legal guardians/next of kin in accordance with the national legislation and institutional requirements. Informed consent was obtained, and patients had the opportunity to decline their clinical records to be used for research.

## Author contributions

JG: Conceptualization, Writing – original draft, Writing – review & editing. YW: Data curation, Investigation, Methodology, Writing – review & editing. HL: Data curation, Methodology, Software, Validation, Writing – review & editing. WD: Data curation, Formal analysis, Investigation, Methodology, Writing – review & editing. WL: Data curation, Investigation, Project administration, Writing – review & editing. CG: Data curation, Software, Writing – review & editing. ML: Conceptualization, Funding acquisition, Resources, Visualization, Writing – review & editing.
